# A shared decision‐making model about care for people with severe dementia: A qualitative study based on nutrition and hydration decisions in acute hospitals

**DOI:** 10.1002/gps.5884

**Published:** 2023-02-07

**Authors:** Kanthee Anantapong, Elizabeth L. Sampson, Nathan Davies

**Affiliations:** ^1^ Marie Curie Palliative Care Research Department UCL Division of Psychiatry University College London London UK; ^2^ Department of Psychiatry Faculty of Medicine Prince of Songkla University Hat Yai Thailand; ^3^ Department of Psychological Medicine Royal London Hospital East London NHS Foundation Trust London UK; ^4^ Centre for Ageing Population Studies Research Department of Primary Care and Population Health University College London London UK

**Keywords:** Alzheimer's disease, caregiver, decision, hospital care, person‐centred care, qualitative research

## Abstract

**Objectives:**

To understand the decision‐making processes regarding eating and drinking for hospital patients with severe dementia and use this data to modify a decision‐making model about care for people with severe dementia.

**Methods:**

From January to May 2021, qualitative semi‐structured interviews were conducted with 29 family carers and hospital staff in England who cared for people with severe dementia during hospital admissions. Interviews were transcribed verbatim and analysed using codebook thematic analysis.

**Results:**

We demonstrated a modified decision‐making model consisting of six stages of the decision‐making process: (i) identify a decision to be made; (ii) exchange information and recognise emotions; (iii) clarify values and preferences of all involved; (iv) consider feasibility of each choice; (v) share preferred choice and make a final decision; and (vi) deliver the decision, monitor outcomes and renegotiation. From this study, decision‐making needed to be shared among all people involved and address holistic needs and personal values of people with dementia and family carers. However, hospital staff often made assumptions about the persons' ability to eat and drink without adequate consultation with family carers. The process was impacted by ward culture, professional practice, and legal framework, which might overlook cultural and personal beliefs of the persons and families. Treatment escalation plans could help inform stepwise treatments, create realistic expectations, and guide future decisions.

**Conclusions:**

Our decision‐making model provides clear stages of decision‐making processes and can be used to guide clinical practice and policy around care decisions for eating and drinking, which is often poorly supported.

## Key points

- Most family carers and hospital staff use best‐interest decision‐making, considering the person with dementia's life history, previous wishes and preferences, current behaviours, and overall health conditions.
- Without adequate discussion with family carers, some staff spoon‐feed or give intravenous fluids to the person with severe dementia because they assume the person had lost their ability to eat by themselves, diminishing their autonomy and functions.
- In the shared decision‐making process, it is essential to empower the person, family members, and staff. It is important to elicit the person's cultural, professional, and personal values, using a clear and consistent protocol and well‐established trust.
- Treatment escalation plans could ensure everyone has a shared understanding of care and treatment.

## 1 INTRODUCTION

People with severe dementia often experience eating and drinking problems, including swallowing difficulties and eating‐related behavioural changes.^1^, ^2^ These can lead to aspiration pneumonia, malnutrition, weight loss, skin breakdown, poor wound healing, and increased confusion.^3^ Hospital admissions may aggravate eating and drinking problems due to the busy hospital environment and routines.^4^ Basic support, such as for eating and drinking, can be suboptimal in acute hospitals.^5^ In acute hospitals, around half of family carers reported that their relatives were left with insufficient supervision during mealtimes.^6^

People with severe dementia who have frequent care transitions, including to and from hospitals, are also more likely to receive artificial nutrition and hydration (ANH), including tube feeding^7^, ^8^; however, current evidence in severe dementia shows that tube feeding does not prolong a person's life, prevent aspiration, lead to better nutritional parameters, nor improve quality of life.^9^

The 2005 Mental Capacity Act in England and Wales assumes someone has capacity to make decisions about their care and treatment, unless proven otherwise.^10^ Nonetheless, it can be difficult for people with severe dementia to fully participate in decision‐making.^11^ Family carers and hospital staff may need to support or make decisions about managing nutrition and hydration. They might feel uncertain about the preferences and wishes of the person with dementia. People living with mild dementia may find it difficult to engage with advance discussions about such problems.^12^, ^13^, ^14^

Most previous studies about eating and drinking for people with dementia only involved practitioners with a nursing background,^15^, ^16^ and few studies explored medical decisions in acute care or inpatient hospital settings.^17^ Studies in hospital settings have explored decision‐making in terms of decisional factors, ethical dilemmas, decision outcomes, and overall experiences.^7^

Therefore, we aimed to gain an in‐depth understanding about decision‐making processes regarding eating and drinking for people with severe dementia during hospital admissions from the perspectives of family carers and hospital staff. We used this data to test and modify a decision‐making model rigorously developed using a systematic review,^7^ in which we synthesised and interpreted the literature and mapped it onto a shared decision‐making model for healthcare decisions, the Interprofessional Shared Decision‐Making (IP‐SDM).^18^ The research questions are:

1. What are the processes and information that family carers and hospital staff use when making decisions about nutrition and hydration for people with severe dementia during acute hospital admissions?
2. What are the experiences and needs of family carers and hospital staff involved in the decision‐making process?
3. What approaches to care for nutrition and hydration do family carers and hospital staff feel are acceptable to support people with severe dementia in acute hospitals?

## 2 MATERIALS AND METHODS

We conducted semi‐structured individual qualitative interviews with family carers and hospital staff in England and adopted the ontological assumption of critical realism and the epistemological approach of contextualism, which assumes that some authentic reality exists to produce knowledge, but the knowledge is influenced by social factors, so we can only partially assess the reality.^19^

### 2.1 Population and participants

We used a mix of convenience and purposive sampling and screened the potential participants against the eligibility criteria in Table 1. For research participation, it was recommended not to include families who were bereaved in the past 3 months to respect a period of mourning.^20^

**TABLE 1:** Eligibility criteria for participants in this study.

Family carers
Inclusion criteria Family member or friend who is next of kin or key decision‐maker for a person with severe dementia (current or bereaved/former) Participants must be able to provide informed consent Participants must be able to read and speak English Participants must be over the age of 18 years
Exclusion criteria Family carers bereaved in the past 3 months
Hospital staff
Inclusion criteria Hospital staff in a caring role, either health or social care, for someone with severe dementia Experienced in providing dementia care and contributing to decision‐making related to nutrition and hydration in acute hospital setting Participants must be able to provide informed consent Participants must be able to read and speak English

### 2.2 Participant recruitment and consent process

#### 2.2.1 Family carers

Due to Covid‐19, we were unable to recruit participants from clinical services and recruited family carers from Join Dementia Research (JDR) and online social media. JDR is an online self‐registration service that enables volunteers with memory problems or dementia, family carers of those with memory problems or dementia and healthy volunteers to register their interest in taking part in research. We also used snowballing methods to aid recruitment.^21^ Participant information sheets and consent forms were emailed to potential participants and the participants were given sufficient time to consider the study.

#### 2.2.2 Hospital staff

We recruited hospital staff online using social media or via known contacts of the research team, and snowballing methods.^21^ We purposively sampled staff with a range of roles in the multi‐disciplinary team making decisions about and providing care for eating and drinking for people with severe dementia.

### 2.3 Data collection methods

From January to May 2021, the first author, with a background in psychiatry and gerontology, conducted semi‐structured interviews with family carers and hospital staff. During the interview, we acknowledged potential power imbalance, especially between the interviewer and family carers, as some participants might not share their full experiences and views, fearing being judged by an interviewer who was a clinician.^22^ Unintentionally, the interviewer might also express his ideas through either verbal or non‐verbal language, and these ideas could impose subsequent responses from participants. However, the interviewer was an experienced qualitative researcher and tried to encourage open and honest discussions by emphasising that we wanted to learn about their lived experiences and needs, so there were no right or wrong answers, and all their views mattered to this study.^23^ The interviewer still facilitated natural interactions with participants, but he kept expressing his judgement at the minimum and noted down any ideas emerging during the interviews to use in the analysis stages.

All interviews were done via online platforms (Zoom or Microsoft Teams) and telephone. The interviews were audio‐recorded. All participants provided electronic consent prior to the interviews. An interview schedule, supplemented with case scenarios, was used to guide interviews, informed by our earlier studies, including the previous decision‐making model.^7^, ^12^ The interview schedule explored decision‐making about nutrition and hydration, specifically within acute hospital situations. We also used case scenarios providing a hypothetical situation that required a series of decisions in acute hospitals (see Supporting Information S1).

### 2.4 Data analysis

All interviews were pseudonymised and transcribed verbatim. Interview transcripts were organised in NVivo version 12. We used both inductive and deductive analytical approaches, and analysed data using codebook thematic analysis, which used some structured and pre‐determined coding framework for developing and documenting the analysis.^24^ The interviews with family carers and hospital staff were analysed using the same coding framework and mapped into the same themes. This was because the interview schedules were overarching and aimed to explore the topics from different perspectives of family carers and hospital staff.

The first author started coding interview data after the first few interviews were conducted, as such, data analysis was an ongoing process and informed subsequent interviews. This process also determined when there were no new codes being used and that the number of participants was enough.

Using a team‐based approach, we iteratively developed codes and themes. We mapped them into the pre‐determined coding framework reflecting six stages of the decision‐making model developed in our previous systematic review.^7^ The previous model was adapted from the IP‐SDM model,^18^ and comprises six stages: (1) identification of decisions to be made; (2) information exchange; (3) values and preferences clarification; (4) consideration of feasibility; (5) preferred choice and actual decision; and (6) implementation, outcomes and renegotiation.^7^

The earlier model was then modified to reflect the current findings and be applicable to other dementia care, through regular discussions among the research team who had clinical and research experiences in old age psychiatry, psychology, gerontology, and dementia and palliative care, with feedback from Public and Patient Involvement (PPI) members.^25^

### 2.5 Ethical considerations

Ethical approval was granted by the Health Research Authority committee of England (Camden & Kings Cross Research Ethics Committee, REC reference: 20/LO/0049).

## 3 RESULTS

### 3.1 Participant characteristics

We interviewed 12 family carers and 17 hospital staff (henceforth staff) (Table 2). Each interview lasted approximately 1 h.

**TABLE 2:** Participant characteristics of the interview study with family carers and hospital staff.

Participant characteristics	Family carers (*N* = 12)	Hospital staff (*N* = 17)
Age (years)		
Mean	53.2	38.9
Range	29–78	28–54
Gender		
Female	10	16
Male	2	1
Marital status^a^		
Married or in a civil partnership	7	9
Single never married/in a civil partnership	3	6
Co‐habiting with partner	1	0
Divorced	0	1
Widowed	1	0
Ethnicity		
Asian/Asian British	2	4
Black (Caribbean)	1	0
White (English, British)	7	11
White (Irish)	0	1
White (other European)	2	1
Family carers characteristics		
Current caring situation		
Bereaved carer	8	‐
Current carer	4	‐
Relationship to the person with dementia		
Daughter/son (caring for mother)	10	‐
Spouse (wife)	1	‐
Friend	1	‐
Hospital staff characteristics		
Professions		
Dietician	‐	1
Clinical psychologist	‐	1
Nurse (*N* = 5)		
• Clinical nurse specialist in older adult	‐	1
• Consultant nurse in palliative care	‐	2
• Lead nurse in nutrition	‐	1
• Ward sister	‐	1
Physician (*N* = 3)		
• General medicine consultant	‐	1
• Geriatrician consultant	‐	1
• Palliative care consultant	‐	1
Speech and language therapist	‐	7
Years in working with older people with dementia		
Less than 1 year	‐	1
1–5 years	‐	5
5–10 years	‐	3
More than 10 years	‐	8

^a^ Missing data.

We label quotes from a dietitian, clinical psychologist and speech and language therapist as from ‘therapy staff’, to maintain the confidentiality.

### 3.2 Key findings

**FIGURE 1:**
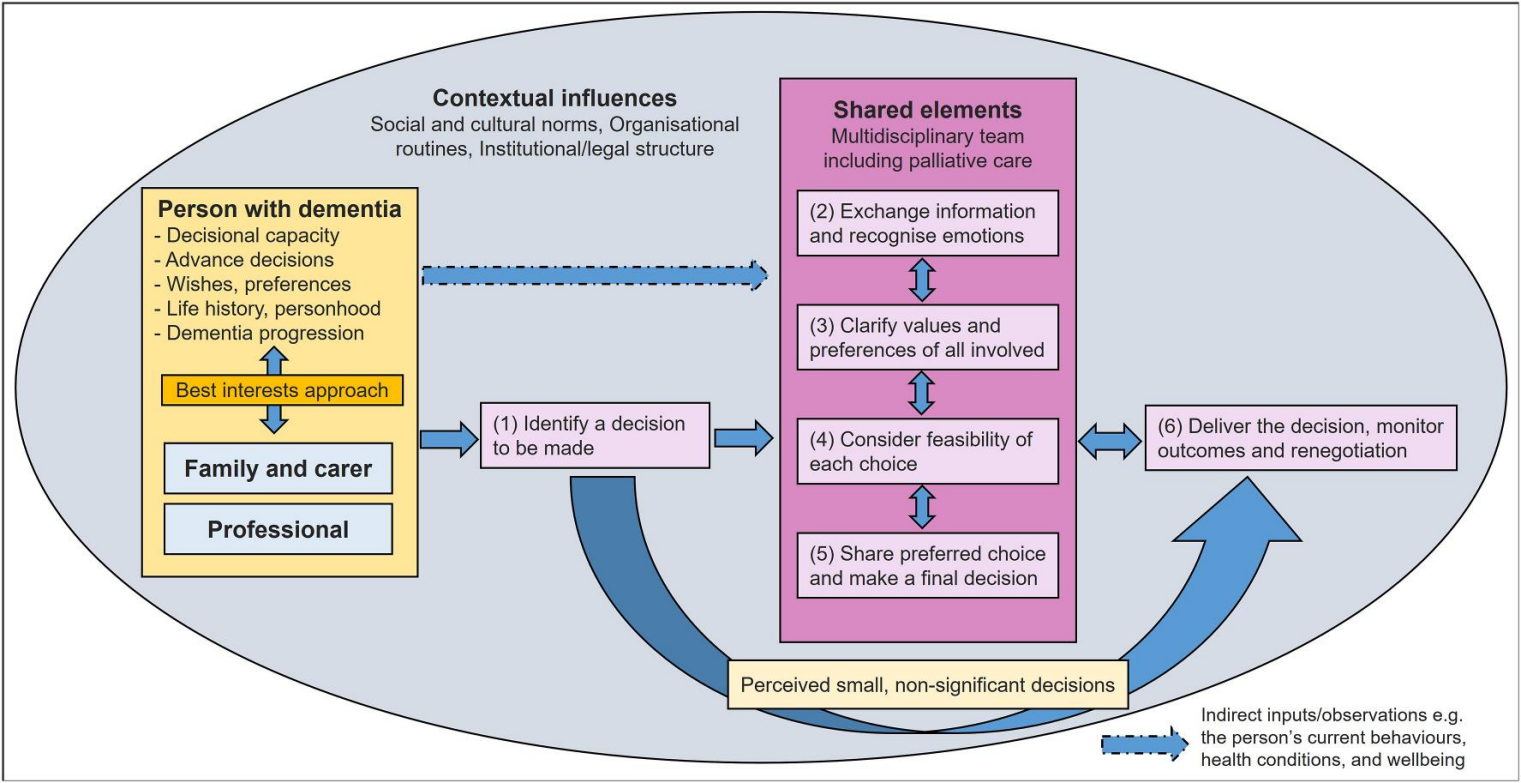
Shared decision‐making model about care for people with severe dementia.

We used our data and modified the previously developed decision‐making model to value best‐interests decision‐making and multidisciplinary approaches (see Figure 1). Our current model further highlights input from the person with dementia in several forms, for example, the person's life history, previous wishes, and preferences (including advance care plan [ACP]), current behaviours, and the overall health conditions. It also emphasises multidisciplinary teamwork and supports the inter‐professional approach in the original IP‐SDM model,^18^ which is not sufficiently recognised in the earlier version.^7^

We developed six overarching themes, which are mapped into the six stages in the modified model (Figure 1) and listed in Table 3. The themes are described narratively below.

**TABLE 3:** Themes mapped into six stages of the decision‐making model.

1. Identify a decision to be made: *Avoiding assumptions about people's eating and drinking*
2. Exchange information and recognise emotions: *Information to meet holistic needs of all people involved*
3. Clarify values and preferences of all involved: *Meaning of (not) eating and drinking for the person and families*
4. Consider feasibility of each choice: *Hospital culture and legal restriction*
5. Share preferred choice and make a final decision: *Sharing responsibility and empowering all decision‐makers*
6. Deliver the decision, monitor outcomes and renegotiation: *Establishing treatment escalation plans*

#### 3.2.1 Identify a decision to be made: *Avoiding assumptions about people's eating and drinking*

Family carers wanted to report to staff that their relative had existing eating and drinking problems before they were admitted to a hospital and would require some support during hospital admissions. Some staff wanted to know the individual's medical history, for example, specifically about prior chest infections, which might suggest existing eating and drinking problems at home or in a care home.

I'll ask them about chest infections, because people don't necessarily pick up on… “Oh, yes they've been coughing for a while when they drink,” and that just becomes completely normal for them, but then… “Oh, yes he's had antibiotics about three times this year.” That's not a protective cough, or maybe related to eating and drinking.

(Therapy staff, PF11)

New eating and drinking problems resulting from acute medical illnesses may develop in hospitals and be noticed later by staff. Otherwise, if the family were allowed to visit and saw the person's food left uneaten, ‘*it was rare that the family don't raise it*’ (Nurse, PF03) to the hospital team.

Hospital environments and constantly changing staff could also cause new eating and drinking problems or worsen existing problems.

Because all these staff are changing shifts regularly… they have a handover regarding medical things, but they wouldn't have a handover in regard to what the person has eaten or not eaten, if the family is not there, then the other staff member may not be aware of what's going on with the patient.

(Former carer, C11)

Family carers felt frustrated if staff did not try to investigate and made assumptions about their relative's preferences and ability to eat or drink. For example, some family carers believed their relatives were spoon‐fed or given intravenous fluids because staff assumed that the person could not eat by themselves, or that staff did not have enough time to provide individual care. This represented the rigid hospital routine and the lack of person‐centred care, diminishing the personhood and independence of the person.

When she came out of hospital, they said to me, “She can't feed herself. She doesn't know how to feed herself.” But I said, “Yes, she always feeds herself – maybe with a little bit of support, but we don't spoon‐feed her.” And they had been spoon‐feeding her at the hospital … I assumed that they were just really busy, and they spoon‐fed her.

(Former carer, C08)

Eating and drinking were not seen as a priority in hospitals because ‘*it's such a natural process*’ and people ‘*[don't] necessarily think about it. [Until] something goes [really] wrong, people then start talking about it or thinking about it more*’ (Therapy staff, PF02). Staff might perceive some eating and drinking decisions were too small and insignificant (as represented by the pale‐yellow block at the bottom of the model in Figure 1) and so adapted the ways they offered food to the person without adequate consultation with family carers or multidisciplinary team.

#### 3.2.2 Exchange information and recognise emotions: *Information to meet holistic needs of all people involved*

Most staff considered eating and drinking problems in the context of the overall progression of the person's dementia, and this should use shared decision‐making processes and required discussions with patients, family carers and multidisciplinary team (see the ‘shared elements’ box in the model). The involvement of the multidisciplinary team can fill knowledge gaps about the person and help communicate between those involved. However, they recognised that other staff in acute hospitals might focus more on treating acute illnesses and did not thoroughly consider the person's underlying dementia.

So, the stroke does get PEG [percutaneous endoscopic gastrostomy], but if they have a history of stroke and then come in with a history of [advanced] dementia… We tend to have a real discussion with the relatives. Saying it's not the stroke which are priority now, it is the dementia side of things…. the tube is not productive anymore. But these are views only of me and not the other clinicians.

(Physician, PF07)

Most staff would assess if the person could make decisions for themselves or express their needs, sometimes by observing the person's behaviours (indirect inputs/observations in the model). They also agreed to check whether there has been prior discussion about eating and drinking problems (see ‘Person with dementia’ in the model). Many staff valued and looked for an ACP and a family member or person with power of attorney; however, this was sometimes overlooked.

So, they rushed him over to [name of hospital] and… he went ahead in the ambulance… by literally within five minutes, they had four cannulas in his arms… they put in a catheter. I thought, ‘crikey, what's going on here?’ [the participant held a power of attorney and considered palliative care for their relative].

(Former carer, C10)

Both family carers and staff emphasised that hospitals should provide holistic care to meet basic needs of the person and eating and drinking formed part of it; however, this was often missed.

I found out that she hadn't been given the eye drops that she was meant to be… she had a leg ulcer that needed…to change the dressing. That also got forgotten. So the last thing they were going to tell me was what she was eating or drinking, because they weren't even coping with the continuation of her care for other needs.

(Current carer, C05)

Whilst gathering information and discussing eating and drinking, staff tried to regularly check family carers' emotions and provide reassurance about the situation. Some staff might experience emotional difficulties in discussing eating and drinking. However, they reported that they gained more confidence over the years, but ‘*it never gets too easy because then [they] might get complacent and it means [they] don't really care*’ (Therapy staff, PF12).

#### 3.2.3 Clarify values and preferences of all involved: *Meaning of (not) eating and drinking for the person and families*

Most carers and staff thought that there was not ‘*one solution [about eating and drinking problems] for everybody*’ (Former carer, C10), and it was important for family carers and staff to help elicit the cultural and personal values about the food and drink preferences of the person with dementia.

[Persons with severe dementia] cannot exercise their authority or their decisions on the medical treatment…. But food is the only thing where they can exercise their authority whilst in the hospital.

(Therapy staff, PF16)

It could be particularly challenging for persons without English as their first language as they might have lost their language ability and could be ‘*not understanding [or speaking] much English anymore*’ (Former carer, C04). This could make the person unable to express their needs about eating and drinking to staff if there was no family to help communicate. This was particularly problematic during the Covid‐19 pandemic.

If death was not imminent, it seemed important for most carers and staff to help the person receive adequate food and fluids because ‘*it's all part of getting somebody well*’ (Current carer, C06). Most family carers and staff wanted to help the person to continue to enjoy eating and drinking by mouth, including offering food based on their religious and cultural background.

Some staff reported they saw ‘*[some families] almost like trying to open [the persons'] mouths quite forcefully*’ (Therapy staff, PF12) in the hospitals. However, every family carer and staff in this study said they would not force the person with dementia to eat or drink because it could be ‘*a bit infantilising, turning [them] into a child*’ (Former carer, C08). Refusal to eat and drink could be seen as how the person was able to express their choice and a point where they ‘*don't want to live [and ask someone to] help [them] die*,’ but participants acknowledged that ‘*[they] can't [do it because] there is no euthanasia for dementia and [they] just have to wait for the time*’ (Former carer, C08). However, they would support other ways for quality of life and dignity throughout the time.

Most family carers thought that if ‘*[people with dementia] are just having [food and fluids] intravenously or through a tube, [we're] taking away part of someone's life*’ (Former carer, C01) or their quality of life. In their views, tube feeding ‘*was just prolonging the inevitable*’ (Current carer, C02). However, most carers and staff acknowledged that it could be emotionally demanding in some cultures where people considered food and drink as the way family expressed love and care for their relative.

If they said, “we are definitely going to put a tube in again”, I would resist…I would be very vocal about that… But some people do have very strong beliefs that they must carry‐on preserving life for as long as possible, and then therefore it must be quite difficult.

(Former carer, C03)

#### 3.2.4 Consider feasibility of each choice: *Hospital culture and legal restriction*

Decisions about eating and drinking are influenced by wider contextual factors, including professional culture, hospital routine and legal frameworks (aligned to the outer circle of contextual influences). For example, although most carers and staff wanted the person with severe dementia to have food and drink by mouth, it is difficult for the person to adapt to the less flexible hospital food menus, mealtimes, and environment.

Sometimes we think like you need to eat three big meals a day when they haven't been doing that at home, they just snack throughout the day, and that's OK… In hospital, it's a bit difficult to work around that when catering in terms of what that person would have at home.

(Nurse, PF05)

Many staff mentioned policies in their hospital to support oral eating and drinking, for example, finger foods, protected mealtimes, and permission for families to come in and help with eating during the mealtimes. However, both family carers and hospital participants mentioned that each ward within the same hospital could have some variations in approach to eating and drinking problems of people with severe dementia. Some family carers reported their relative had been in acute medical wards, for example, Cardiac or Orthopaedic wards. When transferred to Elderly or Dementia wards staff were ‘*more interested in [their relative] as a person*’ (Former carer, C08) and keener in caring for people with dementia, including for eating and drinking.

Staff mentioned that professional culture and practice about eating and drinking problems in people with severe dementia has changed over recent years. They acknowledged the growing evidence about limited benefits and potential harms of ANH and suggested these decisions would now require a clearer clinical and ethical framework to prevent the overuse of ANH.

I've been working in palliative care for 15 years or so now and when I first started doing my training… if somebody might be in their last year of life, we don't use artificial feeding. Not in a blanket rule way but just culturally… It wasn't a question that anybody talked about. Whereas now I would say it's much more conscious discussion and decision‐making.

(Physician, PF04)

Considering the legal restriction and practice guidelines, most staff wanted to make it clear to family carers that eating and drinking decisions are holistic decisions to be made by the clinical team, using the best‐interests decision‐making approach, but in consultation with other key persons such as family members.

#### 3.2.5 Share preferred choice and make a final decision: *Sharing responsibility and empowering all decision‐makers*

When making decisions, staff wanted ‘*to make sure that we're always putting [the person] in the centre*’ (Therapy staff, PF17), and thought it was ‘unfair’ and ‘too stressful’ to put eating and drinking decisions solely on family carers. Staff sometimes needed to mediate between family requests and the best interests of the person with dementia.

Some family carers preferred to be more passive and ‘*would have to trust [staff] to do… what they felt was the appropriate thing*’ (Current carer, C05). However, most family carers would like staff to ‘*give [them] enough information for [them] to be able to make an informed decision*’ (Current carer, C05). They wanted to have an honest and open discussion with staff and still honour their relatives' wishes to reach a mutual agreement on decisions.

I think the person in the hospital who's responsible for noticing and deciding what should be done… then talk to the patient and their caregiver… and explain what the problem is… possible solutions there are and agree the way forward jointly… but I think the patient's wishes should be the ultimate.

(Former carer, C01)

Some specialist staff such as therapists suggested training and empowering generalists or frontline staff to proceed with the decisions that were best for the individual. This is to avoid unnecessary and harmful delays waiting for specialist consultations.

…leaving the patient nil‐by‐mouth – was that really appropriate if your overall decision in the end was for them to continue to eat and drink at risk? They have had two or three days without anything, and that must be quite distressing… I think about empowering the teams to have that knowledge to be able to go forward with that decision… Should it have to wait for speech therapy to assess the swallow?

(Therapy staff, PF02)

Trust and relationships seemed important to many family carers and staff to facilitate discussions and shared decision‐making about eating and drinking.

It's building up a trust, and it can take time. The main thing is to be completely open and not to be confrontational. Put yourself in what they are going through… Going in there thinking, “well, how dare they” and, “I know best”, that's not going to do you any favours at all. But sit down and say, “So tell me about your mum or your dad before all this happened” And that opens them up, because they feel you're actually interested in my loved one as a person.

(Nurse, PF15)

#### 3.2.6 Deliver the decision, monitor outcomes and renegotiation: *Establishing treatment escalation plans*

Staff thought it was important to think ahead when making and implementing decisions. Many staff referred to treatment escalation plans where they had a ceiling of nutrition and hydration treatments for people with severe dementia. For example, if it was clear that the person with severe dementia had long‐term deteriorating eating and drinking problems or was approaching the end of life, neither family carers nor staff would use tube feeding (both nasogastric [NG] tube and percutaneous endoscopic gastrostomy [PEG]) for the person. They would agree on short term intravenous or subcutaneous fluids and risk feeding, where the person is supported to eat orally as long as it does not cause them distress, despite acknowledging the risks of choking and aspiration.

However, people with severe dementia might have been coping well with eating and drinking before, but ‘*something happens acutely, and knocks them off and that can impact their [eating and] swallowing*’ (Therapy staff, PF17). In this case, intravenous infusion seemed acceptable to most carers and staff. Although it was controversial among staff, many staff might consider using temporary NG tubes for potentially reversible eating and drinking problems. Unlike NG tubes, PEG was still opposed by every family carer and staff for this circumstance.

…Sometimes medical teams, they'll say they've got advanced dementia, they're old and they feel that a nasogastric tube isn't appropriate. But you're trying to show that before hospital… they were actually doing very well. So a trial of a [nasogastric] tube may be worth helping them recover and optimise their nutrition.

(Therapy staff, PF17)

Staff wanted to be honest with family carers about the treatment escalation plans because once the team started a temporary ANH intervention, then it could be difficult to withdraw it. Some families might request the intervention again because it had been previously effective, despite the apparent current deterioration.

In hospital, it can happen. You give someone IV fluids and they perk up, the family are, “Brilliant, she's great.” Then they refuse drinks and become dehydrated again. Then the family says, “But you've got to give them IV fluids because that made them better…” I think the doctors might do it once, if it particularly difficult [situations] they might do it twice, but then they will say, “All right, well, this is …” Then they'll have the discussion about end‐of‐life.

(Therapy staff, PF13)

Continuing to post‐discharge care, staff would signpost or link with the community team who provide eating and drinking support to the person with dementia and their family. However, community services might not always be available for people with dementia because of limited resources.

Our dietitian can speak to the community dietetic service if need be, but to be fair, strokes and the other people with neurological diseases itself take on so much of their time that dementia comes way at the bottom.

(Physician, PF07)

Some family carers reported that community support was sometimes later withdrawn as their relative returned to their baseline eating and drinking at home (or care home).

I got her back into her environment and routine, it took me four weeks to get it all going. She was fine. She was stronger than ever and in fact within six weeks, palliative care withdrew. They were coming fulltime to check on her, to prepare, and they could see that she was not end‐of‐life.

(Former carer, C04)

Many family carers mentioned the importance of support for other symptoms, for example, behavioural and psychological symptoms, which can impact the person's eating and drinking. Some staff would discuss this with the multidisciplinary team and organise a holistic care plan to be sure that the person and family could live well with dementia after the hospital discharge. It sometimes included the end‐of‐life care plan about where and how the person with severe dementia might wish to die and be cared for.

## 4 DISCUSSION

To our knowledge, this is the first study to provide in‐depth insights regarding iterative processes of shared decision‐making about nutrition and hydration for people with severe dementia in acute hospitals. It was grounded on the perspectives of both family carers and staff.

We modified a decision‐making model to reflect our current findings. Studies included in the previous systematic review, in which the earlier model was developed, focussed on the roles and needs of family carers and staff in the decision‐making processes and did not report the interprofessional teamwork.^7^ Our modified model now highlights that the wishes, preferences and needs of people with severe dementia should not be overlooked or overridden by the family's requests or staff's pre‐existing attitudes. Multidisciplinary approaches are also included. These emphasise that family carers and staff should work together and always pursue the person's best interests when making decisions.

### 4.1 Shared decision‐making about nutrition and hydration

A recent study found that people with dementia might not always want to participate in shared decision‐making about eating and drinking,^12^ consistent with previous studies in wider patient populations.^26^ At the later stage, their decisional capacity may also be reduced or fluctuant; however, the person could make their views known in different ways, for example, saying or doing something that meant that they did not want to eat at the time. In this study, most staff would observe and talk to the person with dementia before going to their family carers to respect the person's wishes and promote person‐centred care.

However, some staff assumed the person had limited ability to eat and drink and chose a perceived quick solution for eating and drinking, for example, spoon‐feeding and ANH. This could undermine the person's independence and functions if otherwise they could eat and drink by themselves with only some support.

Considering person‐centred care,^27^ family carers can help communicate with the person, and their knowledge about the person's baseline, wishes, and preferences about eating and drinking can guide the decisions.^17^, ^28^, ^29^ However, in acute hospitals staff may override the person's wishes and preferences and refer to the family carer's opinions.^4^, ^30^

It needs to be clear from the outset that many decisions about nutrition and hydration for people who lack capacity are holistic decisions requiring a best‐interests approach.^31^ This may involve the use of risk feeding and decisions to withhold or withdraw burdensome interventions, including ANH. Some families may find this difficult due to cultural beliefs or emotional unpreparedness,^32^, ^33^ which can be overlooked during discussions^34^, ^35^ and lead to conflict between family carers and staff.

### 4.2 Nutrition and hydration support for people with severe dementia in acute hospitals

In acute hospitals, due to unclear protocols, people with severe dementia may be offered a regular diet and fluids or unnecessarily left nil‐by‐mouth during the wait for further investigation or specialist consultation,^31^, ^36^, ^37^ as reported in this study. This can compromise the persons' safety and comfort.

Consistent with previous studies,^31^, ^32^, ^38^ comfort or risk feeding, enhanced social interaction, flexible mealtime, and personalised adjustment to food and drink, utensils, and environment were acceptable to staff and family carers. Many participants recognised that eating and drinking for people with dementia would take time, which could create negative attitudes towards people with dementia as being a nuisance to hospital routine care.^16^, ^39^, ^40^ Family carers and staff perceived each mode of ANH (intravenous fluids, NG tube and PEG) have different degrees of aggressiveness, and this reflected their preferences about ANH for people with severe dementia.

### 4.3 Strengths and limitations

The involvement of both family carers and staff with a variety of backgrounds enhanced the richness of data and provided the creditability of this study. We regularly discussed the research process and findings with feedback from an expert and PPI members to ensure their relevance to clinical practice. Using a team‐based approach, codes and themes were iteratively developed to enhance its rigour.^25^, ^41^

Despite an effort to maximise the variation of participant characteristics, most participants were White, and most family carers were adult children and bereaved carers. In a previous study, compared to non‐spouse carers, spouse carers of people with dementia provided more care and more frequently experienced negative impacts on their social life and psychological and physical health,^42^ which may influence their experiences and needs in providing care for eating and drinking. Bereaved carers might have had some difficulties recalling the details of care in hospital; however, at the time of interviews, all bereaved carers had lost their family member within 1–2 years and still stayed active on the JDR. They would also have time and space to reflect on their decisions and hospital care, providing some significant insights. Due to Covid‐19, participant recruitment and data collection were conducted remotely. This might exclude some eligible participants who may find it more challenging to access Internet or use telephone for the interviews. Some staff were also involved in an initiative to reduce tube feeding in dementia, and many speech and language therapists were keen to participate in this study. Therefore, experiences, cultural values, and needs of participants in this study about eating and drinking and decision‐making may differ from other family carers and staff.

### 4.4 Clinical and research implications

Hospitals and organisations may use our model to produce an agreed risk protocol, considering the overall components of the model. We encourage support for communication and multidisciplinary teamwork that would reconcile the decisions and care plan with the needs and expectations of the people involved.^43^ However, in acute hospitals this can be challenging in practice due to limited time and staff shortage, and this would require national policies and sufficient funding to expand the capacity and lessen these pressures.

Studies exploring real‐time, natural decision‐making are worth pursuing, despite practical difficulties. Our decision‐making model can be applied to future studies on care for people living with similar neurodegenerative diseases. We used this model to develop a decision aid for eating and drinking for people with severe dementia during hospital admissions.^44^ Decision aids may increase knowledge, the quality of communication, and reduce decisional conflict in dementia care.^45^

## 5 CONCLUSIONS

Decisions about eating and drinking in acute hospitals involve whether to encourage oral eating and drinking or consider using ANH. Family carers and hospital staff generally have negative attitudes about tube feeding and would not use it for people with severe dementia. However, in acute hospitals decision‐making processes are often poorly supported and can create frustration to those involved, especially when cultural and personal needs and preferences of the persons are overlooked. From our decision‐making model, best‐interests and a multidisciplinary team approach can help facilitate the decision‐making process.

## AUTHOR CONTRIBUTIONS

Kanthee Anantapong conceived and designed the study, recruited participants, acquired data from interviews with participants, analysed and interpreted data and wrote the manuscript. Elizabeth L. Sampson and Nathan Davies designed the study, supervised data collection and data analysis, contributed to the interpretation of the data and revised and approved the final version to be published.

## CONFLICT OF INTEREST STATEMENT

The authors report there are no competing interests to declare.

## Supporting information

Supporting Information S1

## Data Availability

The data that supports the findings of this study are available in the supplementary material of this article.
