# Supplementary material for: A shared decision‐making model about care for people with severe dementia: A qualitative study based on nutrition and hydration decisions in acute hospitals
Source: Int J Geriatr Psychiatry. 2023 Feb 7;38(2):e5884. doi: 10.1002/gps.5884 (PMC10108087; doi:10.1002/gps.5884)
Supplement: Supplementary file 1 — Supporting Information S1 [file GPS-38-0-s001.pdf]

Supporting information S1

Interview Schedule – Family carers

Part A: Questions and indicative areas

1. **General conversations in a hospital**
   a. Can you give me an example of a time [X] was admitted to a hospital? Why did [X] need to go to hospital? How was [X] doing in a hospital?
   b. What kinds of discussions did you have with the hospital staff about the care and treatment for [X]? Who did you talk to? (may probe with questions in 2c)
2. **Discussion and decisions about eating and drinking on hospital wards**
   a. Did [X] need any help with eating and drinking during the time in hospital? Did hospital staff ask you whether [X] needed any help with eating and drinking?
   b. Have you ever discussed eating and drinking with hospital staff?
      *<u>If no discussion or problems then ask</u> – If hospital staff come to you and say [X] might not eat and drink enough, and [X] might need to change eating and drinking. How do you want to discuss this with hospital staff (or other family members)?
   c. Probe more on how the discussion has been going (or would be going)
      i. Who did start the conversation? Who would you want to talk to? Whether it was up to you to asking help or the staff offer you the help.
      ii. What was your role in the conversation? What would you like to say? Did you have a chance to say? What was decided at the end of that discussion?
      iii. How did you tackle the decisions? whether you involve [X], like their previous wishes? Or prefer to rely more on family and staff's opinions?
      iv. How did you discuss with other family members?
      v. Did [X] have advance decisions? Did it include plan for eating and drinking?
   d. How did hospital staff explain [this information] to you? (or how would you like them to do?)
      i. Prognosis, illness and wellbeing including if [X] not wanting to eat.
      ii. The way we can help improve eating and drinking for [X] (or people with severe dementia)? Did they mention about drips (or tube feeding)? How did they explain about drips and tube feeding?

iii. Did they explain upfront about discharge planning for eating and drinking? How would the eating and drinking treatments be used at home (or a care home)?

e. Influence of personal belief, religious belief, faith, background

i. Are there some other things that may have been going on at the time, for example, your own personal views?

1. How do you think [personal circumstances] would influence or change the way you think about eating and drinking?

ii. Do you think one is more important between eating and drinking? why?

f. When hospital staff changed the way [X] ate or drank (or gave drips/tube feeding), how did they keep you informed about how [X] was doing about their eating and drinking?

i. If the eating and drinking treatments were not helping, how can we rethink about support them with eating and drinking?

ii. How did hospital staff plan for eating and drinking when [X] was discharged? How did you have to change the way you provide care for eating and drinking at home (or a care home)? Was there someone you could go to, to get support for this after they were discharged home/to the care home?

g. (Overall) How did you feel about having these conversations with hospital staff?

i. What made it easier to have these discussions?

ii. What do you think made it harder to have these discussions?

iii. Conversations about eating and drinking in hospitals can be emotional, how can clinical staff help you talk through this topic? (open, sensitive and timely communication)

iv. If no discussion about eating and drinking at all then try – Can you think about other discussions which were quite emotional or sensitive which you have had with staff that went well, what could we learn from that discussion? Do you think to apply to discussions about eating and drinking?

3. We are developing a guide for people like yourself and hospital staff to have a conversation about eating and drinking difficulties. What should this guide look like?

a. What should it include? For example, key facts, types of eating and drinking treatment

b. Who would this help most? How do we get people to use it?

Part B: Scenarios

Scenario 1

Mrs J is 85 and has Alzheimer's dementia for five years. She lives at home with her daughter and her family. Over the last year, Mrs J needs more help and has started to eat less and less. She often refuses to eat. Her daughter has tried many ways to encourage her mum to eat, such as providing suitable foods with careful hand feeding. This has been working. However, it has become more and more difficult over the last 6 months as Mrs J does not swallow food and holds the food in her mouth. It also causes her to choke frequently.

Since last week Mrs J has developed fever and shortness of breath. She is admitted to hospital and diagnosed with pneumonia. When the doctor examines her they find she is very dehydrated and malnourished. Her doctor asks her daughter to decide whether to give Mrs J her liquids via a drip (IV fluids) and then consider feeding her using a tube into her stomach to improve her condition.

Questions:

1. If this were your relative, what would you be thinking? What would be your concerns?
2. How can hospital staff help with your concerns? Who would you go to talk to?
3. In your view, what are the advantages and disadvantages of giving drips? And feeding tube?
4. What would you feel about the situation?
5. What would you like to say? What would you like to know?

Scenario 2

After two weeks of the admission, Mrs J has been treated and discharged to a care home with a tube inserted into her stomach. In the care home, the tube seems to help Mrs J to get enough nutrition as her weight is quite stable. However, she becomes weaker and more unwell. She also looks uncomfortable and tries to pull the tube in her stomach out constantly. Care home staff need to hold her to get the tube in place.

Over the last week, she has developed a fever and is quite confused. She pulls out the tube. She is taken to hospital and diagnosed with a urine infection. When the doctors examine her she also had bedsores on her buttock. Her doctor asks her daughter whether she wants to re-insert the tube or consider just providing small amounts of food she enjoys using small spoons, known as hand feeding and comfort feeding for Mrs J. The doctor also informed that she was quite sick and might be entering her end-of-life time in a few weeks.

Questions:

1. Again, if this were your relative, what would you be thinking about this situation? What would be your concerns?
2. How can hospital staff help with your concerns?
3. What would you feel about the situation?
4. In your opinions, would Mrs. J be suffering if we did not re-insert the tube?
5. What would you like to say? What would you like to know?
6. What are the things that make you feel or think differently from the earlier scenario?

Are there any other topics we have not discussed which you think are important?

Interview Schedule – Hospital staff

Part A: Questions and indicative areas

Experiences of dementia care on eating and drinking in acute hospitals

1. Could you please describe your role in caring for people with severe dementia in hospital?
   a. What kinds of discussions have you had with family carers about the care and treatment for people with severe dementia?

Decisions about nutrition and hydration on acute hospital wards

2. Now I would like you to think of someone with severe dementia who has needed any help with eating and drinking, and you have involved in a discussion about it.
   a. Could you please tell me something about that experience?
   b. Have you ever discussed eating and drinking with carers of the patient? What do these discussions include?

(*Alternative) If people with severe dementia are not eating and drinking enough and you think they might need to change eating and drinking. How would you discuss this with family carers (or other hospital staff)?

   c. Probe more on how the discussion has been going (*or would be going)
      i. Who did start the conversation? Who did you talk to, family or other staff?
      ii. How did you involve in the conversation? What would you like to say?

iii. For multidisciplinary team discussion about eating and drinking, who was involved? Who did lead the discussion? How did you discuss with other hospital staff? What was your role in making the decisions?
iv. How did you tackle the decisions? whether you involve the person with dementia, like their previous wishes? Or prefer to rely more on family and staff's opinions?
v. Did you regularly look for advance decisions? Did they normally include planning for eating and drinking?

d. How do you explain [this information] to family carers? (or how would you like to do?)
i. Prognosis, illness and wellbeing including if the person not wanting to eat
ii. The way we can help improve eating and drinking for people with severe dementia? For, example, any treatment or technique.
iii. How do you explain about drips (or tube feeding) to a family?
iv. Does the discharge plan influence the decisions about eating and drinking?
v. In terms of language, what kind of language do you or your colleagues use? What are the phrases or wording used in these discussions with family?
1. Can you give an example of a good experience or something similar?

e. Influence of personal belief, religious belief, faith, background
i. Are there some other things that may have been going on at the time, for example, your own personal views?
1. How do you think [personal circumstances] would influence or change the way you think about eating and drinking?
ii. Do you think one is more important between eating and drinking? why?

f. When your team change the way people with dementia eat or drink (or give drips/tube feeding), then how do you keep their family informed about how the person is doing about their eating and drinking?
i. If the eating and drinking treatments are not helping, how can we rethink about support them with eating and drinking?
ii. How do you support the person with dementia and their family about eating and drinking when they are discharged? Who do you need to contact for the care at home or a care home?

g. (Overall) How do you feel about having these discussions?
i. What made it easier to have these discussions?
ii. What do you think made it harder to have these discussions?

iii. Conversations about eating and drinking in hospitals can be emotional, how would you like to help family carers to talk through this topic? (open, sensitive and timely communication)

iv. Can you think about other discussions which were quite emotional or sensitive which you have had with family carers that went well, what could we learn from that discussion? Do you think to apply to discussions about eating and drinking?

4. We are developing a guide for hospital staff like yourself and family carers to have a conversation about eating and drinking difficulties. What should this guide look like?
    a. What should it include? For example, key facts, types of eating and drinking treatment
    b. Who would this help most? How do we get people to use it?

Part B: Scenarios

Scenario 1

Mrs J is 85 and has Alzheimer's dementia for five years. She lives at home with her daughter and her family. Over the last year, Mrs J needs more help and has started to eat less and less. She often refuses to eat. Her daughter has tried many ways to encourage her mum to eat, such as providing suitable foods with careful hand feeding. This has been working. However, it has become more and more difficult over the last 6 months as Mrs J does not swallow food and holds the food in her mouth. It also causes her to choke frequently.

Since last week Mrs J has developed fever and shortness of breath. She is admitted to hospital and diagnosed with pneumonia. When the doctor examines her they find she is very dehydrated and malnourished. Her doctor asks her daughter to decide whether to give Mrs J her liquids via a drip (IV fluids) and then consider feeding her using a tube into her stomach to improve her condition.

Questions:

6. If this were your patient, what would you be thinking? What would be your concerns?
7. What would you feel about the situation?
8. In your view, what are the advantages and disadvantages of giving drips? and feeding tube?
9. If you need to talk to family carers about the situation and discuss the decisions, how would you like the conversations to be?
10. What would you like to say? What would you like to know?
11. How could discussions about other sensitive topics be applied to eating and drinking in this situation?

Scenario 2

After two weeks of the admission, Mrs J has been treated and discharged to a care home with a tube inserted into her stomach. In the care home, the tube seems to help Mrs J to get enough nutrition as her weight is quite stable. However, she becomes weaker and more unwell. She also looks uncomfortable and tries to pull the tube in her stomach out constantly. Care home staff need to hold her to get the tube in place.

Over the last week, she has developed a fever and is quite confused. She pulls out the tube. She is taken to hospital and diagnosed with a urine infection. When the doctors examine her she also had bedsores on her buttock. Her doctor asks her daughter whether she wants to re-insert the tube or consider just providing small amounts of food she enjoys using small spoons, known as hand feeding and comfort feeding for Mrs J. The doctor also informed that she was quite sick and might be entering her end-of-life time in a few weeks.

Questions:

7. Again, if this were your patient, what would you be thinking about this situation? What would be your concerns? What would you feel about the situation?
8. If you need to talk to family carers about the situation and discuss the decisions, how would you like the conversations to be?
9. What would you like to say? What would you like to know?
10. In your opinions, would Mrs. J be suffering if we did not re-insert the tube?
11. What are the things that make you feel or think differently from Scenario 1?

Are there any other topics we have not discussed which you think are important?
